# Observational Study on Cardiac Activity in Rescue Dogs with Holter and Electrocardiogram Methodologies during a Simulated Search Activity

**DOI:** 10.3390/ani14121818

**Published:** 2024-06-18

**Authors:** Mirella Lopedote, Annarita Amodio, Maria Ferrara, Francesca Sciutto, Maria Stella Rigo, Giuseppe Spinella

**Affiliations:** 1 Ambulatorio Veterinario «Fisio & Sport», 38098 Trento, Italy; maria_ferrara@outlook.com (M.F.); francesca.sciutto@gmail.com (F.S.); mariastella80@yahoo.it (M.S.R.); 2 Cardio Vet Puglia, 70044 Bari, Italy; cardiovetpuglia@gmail.com; 3 Department of Veterinary Medical Sciences, University of Bologna, 40064 Bologna, Italy

**Keywords:** search and rescue dogs, Holter, electrocardiogram, working dogs, heart, canine

## Abstract

**Simple Summary:**

Working dogs, specifically search and rescue dogs, represent a fundamental resource in the social field for their activities carried out both in daily life and in disaster conditions. Canine well-being must therefore represent an obligation for the governance of a country as well as veterinary clinical research. Our aim was to verify the use of accurate tools for monitoring cardiac activity during operating in the field. The study conducted with electrocardiogram and Holter methods highlighted, in 31 healthy dogs, the presence of few electrical alterations during work with the use of the Holter or, immediately afterwards, with the use of the electrocardiogram.

**Abstract:**

The aim of this study was to observe electric cardiac activity in real working conditions, with the application of Holter and the electrocardiogram in search and rescue dogs. Thirty-one handlers of search and rescue dogs voluntarily participated in this study. Nine dogs were selected to wear the Holter, and twenty-three were submitted to electrocardiographic recordings (one dog, excluded by Holter examination, was then included in the ECG group). Our results showed few cardiac rhythm alterations, such as escape beats, premature ventricular beat, and depression and elevation of the ST segment, particularly during the working phase in the Holter group and during recovery time immediately after activity in the electrocardiographic group. Detected alterations in real working conditions may provide more information than routine checks, and Holter monitoring can be more functional. However, not all dogs tolerate wearing the Holter harness, and more time is thus needed to apply the equipment. In addition, the results are not immediate, and the absence of water is essential because it would damage the equipment.

## 1. Introduction

Physical parameters, particularly cardiac and pulse rates, have been widely documented in working dogs during competition or real-life activity [1,2,3,4]. Working dogs have reported significant increase in pulse rate values before and after competition, compared to the rest status, while respiratory rate values generally increased significantly after the competition. Increases in pulse rate in working dogs were commonly due to the anticipatory response to the competition-related excitement in the pre-activity phase and to physical effort in post-activity phase. Respiratory rate variations in the post-activity phase were mainly related to physical effort and, partially, by environmental temperature and relative humidity [2,4]. More accurate monitoring of cardiac function is generally only performed during specialized clinical examination. Moreover, useful clinical information on cardiac function and activity could also emerge from continuous monitoring even during work activity in the field, in order to better exclude any sub-clinical cardiac dysfunction (i.e., arrhythmia) induced by physical effort. However, this specific information in relation to the entire race track could be obtained only by the continuous cardiac monitoring provided by the Holter method. 

In veterinary sports medicine, the Holter has been proposed to monitor the cardiac function of beagle dogs during treadmill exercises [5,6,7]. In 2020, Restan et al. reported that dogs submitted to endurance-training programs showed decreased resting heart rate and increased time-domain indices [5]. Previously, Swanson et al. in 2019 applied Holter monitoring in thirteen dogs that were walked or trotted on the treadmill to develop a perceived exertion scale [7]. However, all these previous studies have investigated the use of Holter monitoring in specific conditions just to ensure the efficacy of this technique, but, to the authors’ knowledge, there is no available research focused on dogs in real-life working conditions.

The objective of this study was to report the canine cardiac activity with the application of Holter and ECG methodologies in two groups of dogs performing a search activity in the field during a “simulated” missing person. The electrocardiogram (ECG) was recorded at three different times during search activity. 

## 2. Materials and Methods

Thirty-one handlers of search and rescue dogs (SRDs) voluntarily participated in this study. Informed consent was obtained by all owners. The study was correlated to a wide study on sports and working dogs, approved by the Animal Welfare Committee of the University of Bologna (protocol number: ID 4491). Dogs were randomly divided into two groups for the two days of competition. The search took place in two wooden areas of approximately five hectares featuring similar vegetation and ground typology. The only notable difference was the presence of a stream in one of the areas. Twenty-five minutes of search activity was assigned to each dog with the specific focus of locating a “simulated” missing person. 

Before the trial, all dogs were clinically monitored by a licensed doctor in veterinary medicine to verify eligibility to perform the scheduled search activity and to ensure the canine’s good state of health. No dogs received any medication or drugs within 15 days before the simulated activity. Signalments and physiological parameters (respiratory rate (RR), rectal temperature (RT), superficial mucous membranes, capillary filling time) were recorded for all dogs. 

**Figure 1:**
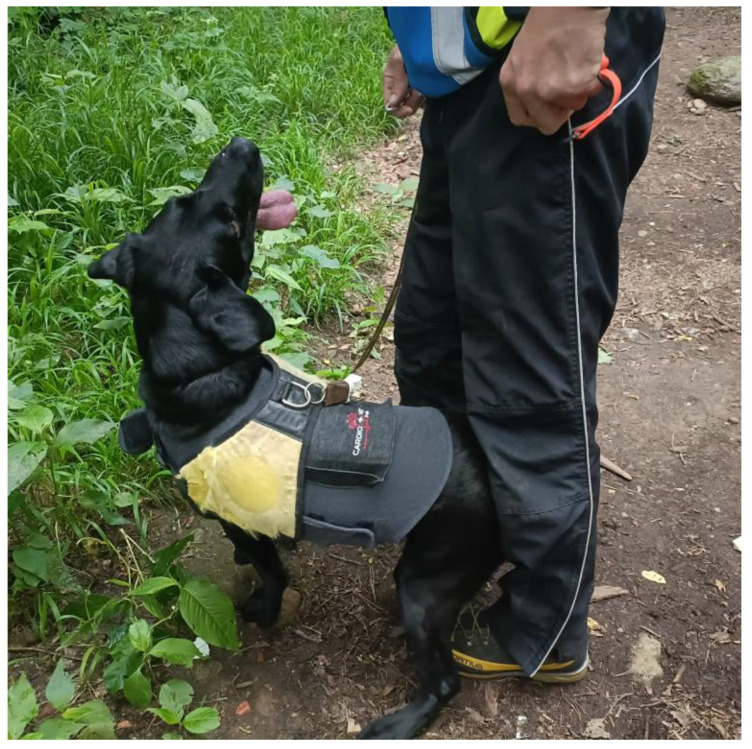
Example of dog with the Holter device contained in the pocket of the stretchy bodysuit.

Nine dogs (five during the first day of study and four during the second day) were selected for the Holter monitoring with five-lead placement (3 on the right and 2 on the left side—Edan Holter System, V1-, Shenzhen 518122, China) for the entire duration of the search activity and for the two hours following it. Dogs in the Holter group were selected for their tolerance to harness wearing, because the presence of the vest, which sustained the equipment, could impact the dog’s search performance if they were not used to working with a harness. Two methods of Holter application were utilized: the first one resulted in being unsatisfactory because, shortly after less than 20 min from the start of search activity, the Holter system was lost with a limited recorded trace affected by several artifacts. In this first method, the trichotomy was carried out only in the area where the five Holter electrodes were applied; it was fixed with soft band and vetrap, and it was covered by a bodysuit that did not well adhere to the animal’s body. Consequently, a second method was adopted, and dogs were previously submitted to a trichotomy in a wide area for electrodes application, previously cleaned with alcohol for better electrode adhesion. Three electrodes were applied on the right hemithorax (red, white, green) and two on the left hemithorax (black and orange). Each dog wore a stretchy bodysuit with a ventral velcro opening and a Holter pocket on the back. After positioning the Holter in the pocket and passing the cables through the slot positioned between the velcro and the pocket, with cables completely hidden by the dress, the Holter was connected to the adhesive electrodes that were secured to the skin with a Tensoplast CA band (Figure 1).

The search area without the water stream was selected for this group in order to avoid any potential water immersion that could have damaged the equipment.

All other twenty-two dogs were submitted to 3 min of 12-leads-ECG recordings (Easy ECG, Contec 8000G\GW—Contec Medical Systems Co., China) before the trial (T0), immediately post- (T1) and 2 h post-activity (T2). 

Both cardiac monitoring methods were applied for examining the cardiac function and rate, along the entire working period for Holter and in three specific phases with ECG application. 

All data were submitted to descriptive statistical analysis (mean ± standard deviation and median) for the entire team (for physiological parameters) and within Groups 1 and 2, specifically for the cardiac observation. 

## 3. Results

Thirty-one dogs (11 males and 20 females), with a mean age of 5.1 ± 2.4 years, were enrolled in the simulated trial. The most represented breeds were Belgian Shepherd Malinois (9), Labrador (7), Golden Retriever (6); other breeds were Australian shepherd dogs, Border collies, and mixed breed dogs. In 15 dogs, the pre-activity mean respiratory rate was 68.3 ± 22.6 breaths per minute; while the other 16 dogs showed polypnea, probably related to the excitatory effect for starting trial. Mean rectal temperature of all dogs, before activity, was 38.5 °C ± 0.15. Apparent mucosae and capillary filling time parameters were within physiological ranges. The environmental temperature was similar for both days: 21 °C with a relative humidity of 76%. 

Mean detected heart rate (HR) with Holter was 117 ± 15.5 beats per minute for the full duration of the application, with a minimum mean of 51.4 ± 10.8 at rest and a maximum mean of 267 ± 47.6 revealed during activity (Table 1). The Holter application highlighted six dogs with sinus rhythm and tachycardia. Moreover, we observed (a) one dog with sinus rhythm and tachycardia that presented one sinus pause followed by one ectopic ventricular escape beat, and (b) one dog with sinus rhythm alternating with sinus tachycardia interrupted by a single ventricular premature ectopic beat.

Only one dog, who wore the Holter system fixed with the first method, reported artefacts during the whole trace: this dog was excluded from the Holter evaluation and was then included in the ECG group. 

The HR evaluated with the ECG showed mean values in T0 of 107.7 ± 37.1 beats per minute, 121 ± 25.2 in T1 and 99.4 ± 21.1 in T2. For two dogs (numbers 3 and 12) and one dog (number 3), ECG evaluation was not performed in T1 and T2, respectively (see Table 2).

In T0, 13 dogs presented a sinus rhythm and 9 dogs a sinus arrhythmia. 

In T1, we found (a) 12 dogs with a sinus rhythm; (b) five dogs with a sinus arrhythmia; (c) two dogs (6-year-old and 4-year-old males Malinois) with a sinus tachycardia, (d) a 1-year-old intact male Golden retriever dog with a sinus arrhythmia with ST-segment depression, (e) a 7-year-old old intact female Labrador dog with a sinus rhythm with left mean electrical axis deviation, and (f) a 10-year-old female spayed Golden Retriever dog with sinus rhythm and occasional ST segment elevation just outside the range (0.25 mv). In T2, 15 dogs presented a sinus rhythm and 7 had sinus arrhythmia. 

## 4. Discussion

The aim of this study was to evaluate potential alterations of cardiac activity in dogs submitted to real-life working conditions through Holter or ECG application. Studies reported in the current literature have always been carried out either in conditioned environments or in competition, but generally they were conducted on endurance disciplines such as sled dogs, or sprints such as greyhound racing or agility competitions. Search and rescue dogs fall into medium-intensity sporting activities with specific resistance and speed needs because their work combines different disciplines of different intensity and physical effort (running, obedience, transport, etc.). Consequently, we decided to report the cardiac electric activity on this not previously investigated category through the application of two different methodologies (Holter and ECG) in order to detect any potential abnormality that could develop during real search activity. 

Our results showed few cardiac rhythm alterations, particularly during the working phase in the Holter group and during recovery time (T1) in the ECG group.

Heart rate (HR) has historically been used to evaluate physical status and effects of type and intensity of exercise; however, HR variations may reflect other physiological or paraphysiological status such as conditioning, fear, anticipation, and pain. Therefore, HR data may be used during training sessions to evaluate the dog’s efforts, cardiovascular status, pain, and recovery time [6]. This observation has great value in working dogs because their activity is strongly influenced by various environmental variables such as temperature, humidity, wind speed, and physical and mental stress. For this reason, if cardiological checks are carried out in a neutral environment, such as a vet clinic, the obtained results do not always correspond to real condition. In a study performed by Vazquez et al. in 1998, the authors observed that physical exercise may induce a significant increase in cardiac arrhythmias in athletic dogs, above all in three specific circumstances: when warm-up is inadequate, when the dynamic exercise approaches maximum capacity, and during the initial recovery period [8]. A similar condition probably occurred in our study. 

The study, conducted by Vazquez et al., in 1998 also investigated the incidence of cardiac arrhythmias following maximal dynamic exercise of racing greyhounds as a model. The study was conducted on 399 greyhounds that participated in scheduled speed races in tracks of 350 or 425 m. Two electrocardiograms were recorded in each animal: the first one before participating in the race and the second one between 90 and 150 s after the race. The observation period was of 5 min. The results showed 112 cases of abnormal arrhythmias, 32 before and 80 after the race [8].

In 2010, Rovira et al. investigated heart rate (HR) and electrocardiographic characteristics in agility dogs compared to values described for sedentary, sled-trained, and racing dogs to determine electrocardiographic characteristics and the incidence of arrhythmias during exercise and recovery. Authors reported that the agility competition leads to a cardiovascular load of 71.67% of the maximal HR. Agility dogs had lower P- and R-wave voltages and shorter QRS and QT durations, but higher PQ duration than other canine athletes (sled dogs and greyhounds). All animals showed vagal-induced arrhythmias before competition, and their incidence decreased during the first 15 min of recovery. None of the dogs developed pathological arrhythmias [9].

The idea of observing and monitoring cardiac activity with the Holter system, and not only with the application of the ECG technique, was related to the main peculiarity of the Holter of monitoring the heart rhythm for a prolonged period during the entire sporting activity. On the other hand, Holter requires more time to apply the electrodes as a good shaving of the hair and good cleansing of the skin is critical for obtaining a better recording. Furthermore, it will be necessary to apply a cover to protect the electrodes, cables, and the recorder, and not all patients can tolerate it [10]. A further strong point to support on the advantage of an adequate and prolonged observation of cardiac activity derives from the wide use of Holter application during physical exercise in horses and humans to predict energy expenditure and cardiovascular functionality in order to design individual training programs to select the fittest athletes and to detect overtraining conditions [11]. Moreover, continuous monitoring before, during, and after the dog’s activity can help to set up specific and individualized training programs based on the characteristics and responses of each individual athlete.

Dogs’ responses to training exercise are rarely monitored using physiological variables, and cardiac autonomic regulation (CAR) is a relevant determinant of adaptation to resistance training. Efficient training provides a stimulus to increase performance and continuous monitoring of athletes, which may provide data that could be used for scheduling a correct training intensity. So far, common monitoring tools were also blood biomarkers, such as blood lactate concentration, and HR to monitor the state of the autonomic nervous system.

Regular exercise may interfere with CAR in dogs, as measured by HR and vagal-derived heart rate variability (HRV) indices. Rarely, scheduled training programs are based on lactate threshold to determine HRV using a 24 h Holter analysis [5].

Furthermore, as reported by Rovira et al. in 2010, further factors are involved in the HR response to exercise, such as the intensity and duration of the relative exercise, hydration and electrolyte status, performance, training level, and subclinical diseases that may be highlighted only by ECG examination [9]; however, in our opinion, more useful results could be obtained with application of the Holter. 

A practical aspect, which in our opinion allows us to accurately evaluate the data provided to us by the Holter recording, is to instruct the handler to record detailed information in a “diary” on type of route taken during the search activity, if any occasional events have occurred or changed in the chosen route, the lengthening of the search time due to any potential traumatic events of the dog, distractions due to the presence of wild animals, and interruption of rest time for various causes. 

From the comparison of the data obtained both with Holter and ECG, we found that the majority of dogs presented a sinus rhythm, i.e., a physiological rhythm, during the pre-activity phase and during and after competition, both with Holter and ECG. Nine dogs submitted to ECG examination presented a sinus arrhythmia during and after the activity. This arrhythmia is frequently observed in dogs due to the prevalence of the parasympathetic system [12]; usually sinus arrhythmia is absent in dogs up to 6–8 months because the development of the autonomic nervous system is not completed. However, dogs included in our study were older than one year. A one-year-old entire male Golden Retriever dog showed a sinus arrhythmia with ST-segment deviation in the recording during the T1 of ECG. The ST segment deviation in an electrocardiographic tracing during physical activity could have clinical relevance because it could result from myocardial damage due to cardiac problems and extracardiac diseases. The ST segment is commonly isoelectric under normal conditions (a period in which all ventricular cells have been depolarized and are preparing for ventricular repolarization), although it may present mild variations. Subsegment depression is usually observed during physical exertion and ischemic heart disease is the most frequent cause of ST segment abnormalities.

A multicenter retrospective study conducted by Romito et al. in 2022 aimed to evaluate the electrocardiographic characteristics of the ST segment and explored the potential influence of variables such as sex, age, body weight, and somatotype on this electrocardiographic parameter, as the characteristics of this segment are poorly characterized [13]. The obtained results showed ST-segment deviation in 43 dogs out of 180 healthy dogs. This study led to the hypothesis that mild ST segment deviation is not an uncommon finding, ST segment depression is more common than elevation. ST segment depression and elevation have similar amplitude that are frequently, but not always, less than 0.2 mV; horizontal and concave morphological patterns are over-represented compared to other morphologies in dogs with ST-segment depression and elevation, respectively. The investigated variables (sex, body weight, age and somatotype) did not significantly influence the probability of developing ST segment deviation. Consequently, application of continuous monitoring, such as the Holter system, during physical activity could be useful for the early diagnosis of myocardial suffering [13]. 

An interesting result has been observed during our Holter examination of a 7-year-old Labrador with an ectopic beat. Ventricular ectopic beats are caused by spontaneous depolarization of an ectopic pacemaker located anywhere in the ventricular specialized conduction system or working myocardium [14]. If the beat originates from the working myocardium, impulse conduction will occur via cell-to-cell depolarization, which is a slow process and will depolarize the ventricles asynchronously. For this reason, the beat is wide and bizarre. If the beat originates within or close to the specialized conduction system, the QRS may even appear normal, making it very difficult to distinguish it from a supraventricular beat [13]. This condition is called premature ventricular beat when it occurs earlier than expected based on the preceding sequence of R-R intervals. They are called escape beats if they occur after a long pause. Common structural cardiac diseases, which may show ventricular arrhythmias, include cardiomyopathies, myocardial trauma, myocarditis, pericarditis, myocardial ischemia, congenital heart disease, and cardiac neoplasia. Among systemic diseases that frequently trigger ventricular premature beats, there are gastric dilation-volvulus, splenic tumors or torsion, pleural effusion, pyometra, prostatitis, pancreatitis, hyper- or hypothyroidism, anemia, uremia, endotoxemia, diabetes mellitus, stress, and anxiety [15]. The observation of an ectopic beat in a dog of our study could be due to the dog’s intensive physical activity, but its presence allowed us to suggest to the handler to carry out further investigations to verify the causes of this detection.

Therefore, some technical limitations have been raised during the Holter application: first, the dog selection, because not all dogs could tolerate the harness during activity, and second, selection of a field without water sources as the Holter system can get wet but cannot be submerged. In addition, the results obtained by Holter are not immediately interpretable as it is necessary to download the traces and results have to be analyzed by a trained cardiologist. On the contrary, the advantage of the ECG has been in the immediate interpretation of the results during the exam; however, technical limitations of the ECG methodology were the selection of the location for the exam and the impossibility to carry on continued monitoring during the activity. In addition, the team observed less tolerance towards applications of crocodile electrodes three times and lateral recumbency, which can be stressful for some dogs immediately after an operative activity. Another limitation was related to the impossibility of using the two techniques on the same dogs for an adequate comparison between the two methods. In the future, an experimental study comparing the two techniques on the same dogs would be desirable.

## 5. Conclusions

On the basis of our results and after a careful selection and preparation of the patient, ECG and Holter methodologies could provide useful information on canine cardiac activity during work; however, the Holter application additionally offers continuous monitoring during the whole surface search activity. Therefore, the Holter system was well tolerated by dogs used to harness both during the activity as well as at rest; however, its adequate anchorage is mandatory in order to obtain a satisfactory trace for subsequent clinical interpretation by a veterinary cardiologist.

## Figures and Tables

**Table 1:** Signalments and cardiac findings during Holter examination.

N° Dog	Breed	Sex	Age (Years)	Minimum Recorded Cardiac Rate (beats/min)	Max Recorded Cardiac Rate (beats/min)	Cardiac Activity Findings
1	Belgian Malinois	F	5	78	142	Artifacts (excluded dog)
2	Belgian Malinois	M	4	47	284	Sinus rhythm/sinus tachycardia
3	Labrador retriever	M	7	46	283	Sinus rhythm/sinus tachycardia
4	Border Collie	F	8	45	279	Sinus rhythm/sinus tachycardia
5	Australian sheperd dog	F	2	50	295	Sinus rhythm/sinus tachycardia
6	Belgian Malinois	F	6	58	290	Sinus rhythm/tachycardia Interrupted by a pause followed by an escape beat
7	Border Collie	F	8.5	50	267	Sinus rhythm/sinus tachycardia
8	Belgian Malinois	M	10	44	287	Sinus rhythm/tachycardia Interrupted by single premature ventricular ectopic beat
9	Australian Shepherd dog	F	3	45	279	Sinus rhythm/sinus tachycardia

F = female; M = male.

**Table 2:** Signalments and cardiac findings during ECG examination.

N° Dog	Breed	Sex	Age (years)	Cardiac Rate (beats/min) at T0	Cardiac Rate (beats/min) at T1	Cardiac Rate (beats/min) at T2
1	Labrador retriever	F	4	138	131	135
2	Belgian Malinois	M	4	190	170	106
3	Australian Shepherd dog	F	1	152		
4	Labrador retriever	M	4	80	120	90
5	Mixed breed dog	F	2.5	100	120	110
6	Chesapeake retriever	F	7.5	90	150	80
7	Bergamasco sheepdog	F	4	122	89	85
8	Labrador retriever	F	4	80	120	100
9	Golden retriever	M	1.5	80	66	80
10	Mixed breed dog	F	7	80	103	81
11	Belgian Malinois	M	6	184	130	118
12	Labrador retriever	F	7	80		70
13	Golden retriever	F	5	90	135	100
14	Golden retriever	M	1.5	60	77	103
15	Belgian Malinois	F	2	150	141	120
16	Border collie	M	8.5	120	140	90
17	Labrador retriever	F	5	96	110	90
18	Belgian Malinois	M	3	92	105	83
19	Belgian Malinois	F	7	162	160	163
20	Golden retriever	F	10.5	93	125	84
21	Labrador retriever	M	7	80	120	90
22	Golden retriever	F	5	80	120	100
23	Belgian Malinois	F	5	80	110	110

F = female; M = male; ECG = electrocardiogram; T0 = before the trial; T1 = after the trial; T2 = 2 h after the trial.

## Data Availability

All data are contained in the manuscript and were collected by Dr. Mirella Lopedote. Information on ECG and Holter traces are also available on request (mirella.lopedote@yahoo.com).
